# Surgical Resection of Bertolotti Syndrome

**DOI:** 10.31486/toj.21.0012

**Published:** 2022

**Authors:** Gonzalo Sumarriva, Brandon Cook, Paul Celestre

**Affiliations:** ^1^ Department of Orthopedics, Ochsner Clinic Foundation, New Orleans, LA; ^2^ The University of Queensland Faculty of Medicine, Ochsner Clinical School, New Orleans, LA

**Keywords:** *Congenital*, *lumbosacral region*, *pain*, *spine*

## Abstract

**Background:** Bertolotti syndrome is the association of lumbosacral transitional vertebrae and low back pain or sciatica. Lumbosacral transitional vertebrae are vertebrae with large transverse processes that (1) articulate or fuse with the sacrum or ilium and (2) have a caudal disc space. Bertolotti syndrome is relatively common, with an incidence of 4.6% to 7% in patients with low back pain. The exact etiology of Bertolotti syndrome remains uncertain, although several hypotheses have been proposed.

**Case Report:** A 17-year-old male presented with a long history of low back pain refractory to conservative treatment including medications, activity modification, and physical therapy. Unilateral Bertolotti syndrome was suspected. The diagnosis was confirmed with bupivacaine injection at the transitional articulation. The patient was treated with surgical resection of his enlarged left-sided L5 transverse process, resulting in complete resolution of pain.

**Conclusion:** Lumbosacral transitional vertebrae are relatively common, so Bertolotti syndrome should be on the list of differential diagnoses for low back pain.

## INTRODUCTION

Mario Bertolotti first described the association of lumbosacral transitional vertebrae (LSTV) and low back pain in 1917.^1^ LSTV have large transverse processes that may fuse or articulate with the sacrum or ilium, forming a transitional articulation. LSTV are relatively common, with an incidence of 4% to 30%.^2,3^ A transitional articulation with corresponding low back pain or sciatica has been termed Bertolotti syndrome. The incidence of Bertolotti syndrome in patients with low back pain is between 4.6% and 7%.^3,4^

Bertolotti syndrome can be challenging to diagnose given the numerous causes of low back pain. Adding to the difficulty of diagnosis, the exact etiology of how Bertolotti syndrome causes pain remains debated. Bertolotti syndrome can present at any age but typically presents in relatively younger patients.^4^ If left untreated, Bertolotti syndrome may adversely affect activities of daily living and recreational activities. Treatment options include nonsteroidal anti-inflammatory drugs (NSAIDs), physical therapy, injections, radiofrequency ablation, and surgery.

We present the case of a patient with Bertolotti syndrome and outline the successful surgical management of the condition.

## CASE REPORT

A 17-year-old male presented to the orthopedic clinic with a 2-year history of left-sided low back pain that he rated as 8 of 10 on the visual analog scale. Pain was most notable with twisting motions, such as hitting a baseball. The patient was initially seen by his pediatrician who referred him to an outside nonoperative orthopedic specialist 2 years prior to our presentation. Workup included radiographs and magnetic resonance imaging (MRI). Transitional anatomy was not mentioned at that time, and MRI was unremarkable. The patient was diagnosed with muscular strain as the etiology of his back pain. Conservative measures included a course of physical therapy, NSAIDs, muscle relaxers, and oral pain medications. Each of these treatments helped, but pain remained significant with activities. Activity modification was the most effective treatment, and the patient did not participate in sports. The patient subsequently moved and was seen by a new pediatrician. The new pediatrician referred the patient to our orthopedic clinic for treatment.

**Figure 1.**
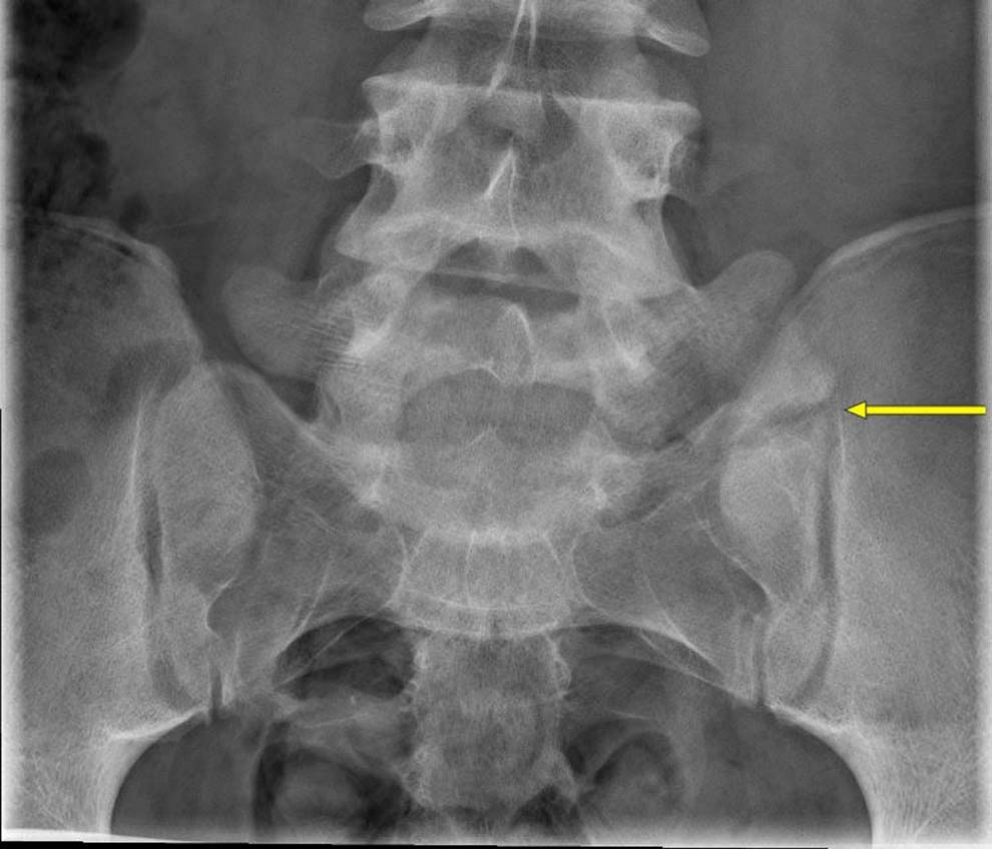
Anteroposterior lower lumbar spine preoperative radiograph demonstrates prominent L5 transverse processes bilaterally (left greater than right). The left transverse process appears to articulate with the sacrum (arrow demonstrates articulation).

On physical examination, the patient had a normal gait. The spine had normal range of motion and no global imbalance. Strength, sensation, and deep tendon reflexes were normal and symmetric to bilateral lower extremities. Pain was re-created with simultaneous lumbar extension and left lateral flexion. Plain film radiographs revealed an L5 vertebra with prominent transverse processes bilaterally, with the left transverse process articulating with the sacrum (Figure 1). MRI without contrast showed no central or foraminal stenosis and no disc herniation. Based on low left-sided lumbar pain made worse with extension and twisting motions, history of failure with conservative measures, and transitional vertebra on radiographs, Bertolotti syndrome was suspected.

A diagnostic left transitional articulation injection of 10 mL of 0.5% bupivacaine under fluoroscopy provided complete pain relief for 8 hours. The diagnosis of Bertolotti syndrome secondary to transitional articulation was confirmed by the complete resolution of symptoms after the diagnostic bupivacaine injection. The Bertolotti syndrome was classified as type IIA under the Castellvi classification,^5^ with the unilateral left transverse process articulating with the sacrum. Surgical treatments, including lumbar fusion vs transitional articulation resection, were discussed, and the patient elected resection.

**Figure 2.**
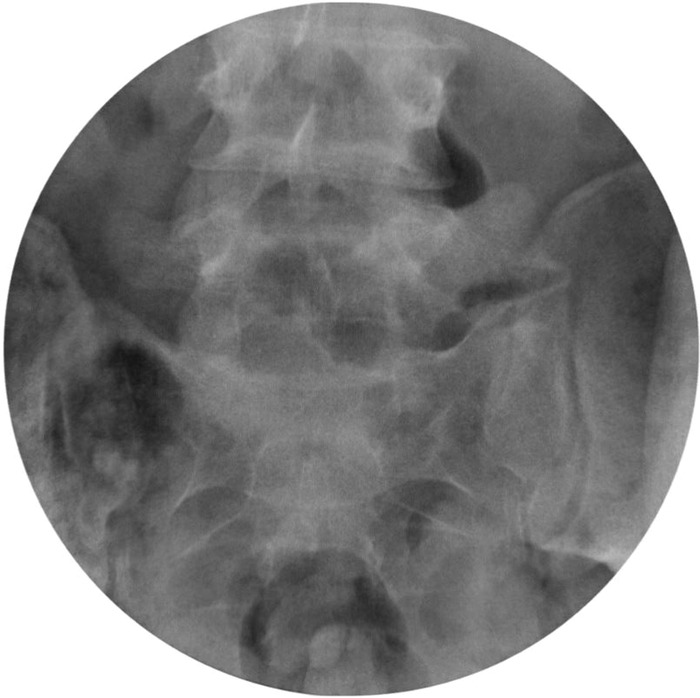
Anteroposterior lower lumbar spine intraoperative radiograph demonstrates excision of L5 transitional articulation with the sacrum on the left.

Using a combination of rongeurs, a high-speed drill, and osteotomes, the left transitional articulation was resected under fluoroscopy. Approximately 7 mm of hypertrophic left transverse process was resected (Figure 2), which was considered to be a sufficient gap to prevent osseous bridging between the transverse process and sacrum postoperatively. The patient was allowed to ambulate as tolerated postoperatively but was advised to avoid heavy lifting and sports. The patient ambulated 400 feet and was discharged on postoperative day 1 with multimodal pain medications, including indomethacin, methocarbamol, and oxycodone-acetaminophen. No postoperative physical therapy was ordered, as the patient had mobilized well in the hospital. At 6 weeks, the patient was allowed to return to sports. At follow-ups, he was doing well, with no pain at 6 months. In a phone interview 4 years postoperatively, the patient continued to endorse no pain.

## DISCUSSION

The exact etiology of Bertolotti syndrome remains debated. Some believe that the pain stems from degenerative and biomechanical changes at the transitional articulation.^6^ Other authors believe that the transitional articulation between the transitional vertebra and the sacrum may limit motion and change the dynamics of the intervertebral discs adjacent to the transitional vertebra. Elster discovered that disc bulges were 9 times higher at the level immediately above the transitional vertebra.^3^ Aihara et al postulated that less degenerative changes occur below transitional vertebrae because the anomalous articulations restrict the motion between L5 and S1.^7^ Other authors do not believe that LSTV and back pain are associated.^8,9^ The controversy about etiology and even the existence of Bertolotti syndrome contributes to the difficulty in diagnosis.

**Figure 3.**
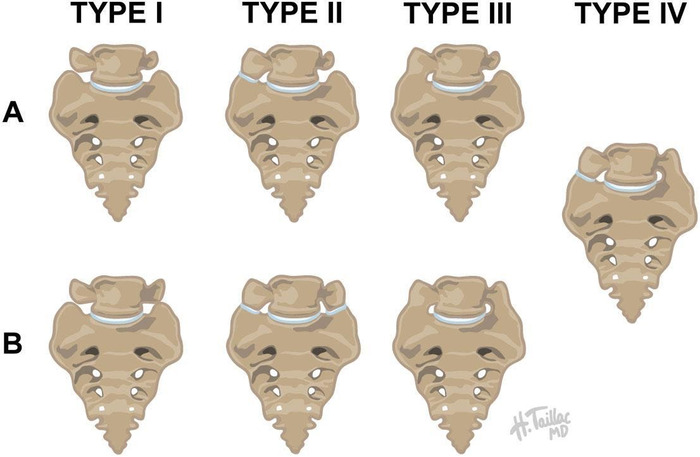
**Transitional vertebrae as classified by Castellvi et al.^5^ Type I has a large transverse process. Type II has a diarthrodial joint between the transverse process and sacrum/ilium. Type III has a bony union between the transverse process and sacrum/ilium. Type IV has unilateral type II and contralateral type III defects. For types I to III, subset A signifies a unilateral defect and subset B signifies bilateral defects.** (Original art by Heather Taillac, MD, Ochsner Clinic Foundation, New Orleans, LA. Published with permission.)

Castellvi et al categorized transitional vertebrae based on the type of connection each vertebra had to the sacrum or ilium (Table, Figure 3).^5^ Type I defects have a large transverse process >19 mm. Type II defects have a diarthrodial joint between the transverse process and sacrum and/or ilium. Type III defects have bony union between the transverse process and sacrum and/or ilium. Type IV defects have a unilateral type II defect and contralateral type III defect. Types I, II, and III can be subdivided into subset A with a unilateral defect and subset B with bilateral defects. In a study of 841 patients with LSTV, Nardo et al found that types I (41.7%) and II (41.4%) were most common, and types III (11.5%) and IV (5.2%) were less common.^10^ Our patient had a type IIA transitional vertebra with a left-sided unilateral transverse process with articulation with the sacrum.

**Table.** Classification of Lumbosacral Transitional Vertebrae by Castellvi et al^5^

Type	Definition
I	Large transverse process >19 mm
II	Diarthrodial joint between the transverse process and sacrum/ilium
III	Bony union between the transverse process and sacrum/ilium
IV	Unilateral type II and contralateral type III

Note: For types I, II, and III, subset A signifies a unilateral defect, and subset B signifies bilateral defects.

Diagnosis of Bertolotti syndrome may be difficult. Differential diagnosis for back pain is broad and includes myofascial pain, sacroiliac pain, fracture (including spondylolysis), spondylolisthesis, scoliosis, disc degeneration/herniation, infection, and malignancy.^4,6^ This broad differential diagnosis likely contributes to delayed or missed diagnoses. Delayed diagnoses likely adversely affect patients’ quality of life through pain and activity restrictions, as was the case for our patient. Workup typically starts with radiographs that demonstrate transitional anatomy. MRI may outline degenerative disc changes or disc herniations that may be more common in patients with Bertolotti syndrome than in patients without transitional anatomy.^6^ Computed tomography may identify stenosis and osteophytes.^6^ Anesthetics or steroids can be injected at the transitional articulation to both diagnose and treat the pain. Injections can help diagnosis by confirming transitional articulation as the site of pain, although no study has confirmed the prognostic value of injections.^6^ Because transitional articulation injections are relatively low-risk procedures, the authors’ opinion is that diagnostic injections should be strongly considered prior to surgical treatment. One factor that sparks consideration for Bertolotti syndrome is younger age. Quinlan et al found that patients with Bertolotti syndrome were typically younger (mean 32.7 years, range 15 to 60 years) compared with patients with single-level disc degeneration (mean 42.6 years, range 15 to 75 years) and multilevel disc degeneration (mean 51.6 years, range 17 to 90 years) based on MRI findings.^4^

The optimal treatment for Bertolotti syndrome is still under investigation and remains debated.^4,6^ Treatment options in the literature include conservative therapy, local injection, radiofrequency ablation, and surgery. Conservative therapy, including physical therapy and NSAIDs, is recommended initially.^6^ Injection can subsequently be used for diagnostic and therapeutic purposes. Almeida et al used radiofrequency ablation before surgical resection in 5 patients and found that 3 patients had only partial pain control.^6^ Two patients had significant improvement after radiofrequency ablation and were completely asymptomatic after surgical resection.^6^

Surgical intervention, which can include resection of the transitional articulation or posterolateral fusion, may be considered after failure of conservative management. Santavirta et al studied 16 surgically treated patients; half received transitional articulation resection and half received posterolateral fusion.^9^ Postoperatively, 10 patients had improved low back pain, and 7 of the 10 had no back pain. The study findings suggest operative treatment for select patients with Bertolotti syndrome: (1) transitional articulation resection for patients with refractory chronic pain truly associated with the transitional joint and no disc degeneration and (2) posterolateral fusion for patients with degeneration in the disc below the transitional vertebra. Three of the 8 patients who underwent posterolateral fusion required repeat operations (2 for nonunion and 1 for disc pathology at the transitional disc level), and 3 of the 8 patients who underwent transitional articulation resection required repeat operations (1 for disc pathology at the level proximal to the transitional vertebra, 1 for repeat resection, and 1 for fusion).^9^

Because our patient failed nonoperative treatment, responded to a transitional articulation injection, and had no substantial disc degeneration noted on MRI, the senior author of this paper felt that transitional articulation resection was the best surgical treatment for the patient. Complications associated with surgical treatment include the risks of any spine surgery (neurovascular injury, dural tear, thromboembolism, infection, anesthesia complication), continued pain, and reoperation.

## CONCLUSION

Bertolotti syndrome is the association of low back pain or sciatica and a lumbosacral transitional vertebra that articulates with the sacrum or ilium. The pain is postulated to be secondary to degenerative or biomechanical changes. Treatment for Bertolotti syndrome remains controversial. Surgical options include transitional articulation resection and/or posterolateral fusion, and optimal surgical treatment requires attention to each patient's unique pathologic anatomy. LSTV are a relatively common finding; therefore, Bertolotti syndrome should be on the list of differential diagnoses for low back pain.

## Notes

*This article meets the Accreditation Council for Graduate Medical Education and the American Board of Medical Specialties Maintenance of Certification competencies for Patient Care and Medical Knowledge.*
